# Localization Matters: Impacts of PEG‐J Localization in Intestinal Levodopa Therapy for Parkinson's Disease

**DOI:** 10.1002/mdc3.14352

**Published:** 2025-02-04

**Authors:** Philipp Klocke, Moritz A. Loeffler, Idil Cebi, Karl‐Ernst Grund, Christine Daniels, Jens Volkmann, Jiri Koschel, Wolfgang H. Jost, Kazimierz Logmin, Lars Wojtecki, Christoph R. Werner, Daniel Weiss

**Affiliations:** ^1^ Centre for Neurology, Department for Neurodegenerative Diseases, and Hertie‐Institute for Clinical Brain Research University of Tübingen Tübingen Germany; ^2^ Centre for General Surgery, Department for Surgical Endoscopy University Medical Centre Tübingen Tübingen Germany; ^3^ Department of Neurology University Hospital and Julius‐Maximilians‐University Würzburg Germany; ^4^ Parkinson‐Klinik Ortenau Wolfach Germany; ^5^ Department of Neurology and Neurorehabilitation Hospital Zum Heiligen Geist, Academic Teaching Hospital of the Heinrich‐Heine‐University Düsseldorf Kempen Germany; ^6^ Institute of Clinical Neuroscience and Medical Psychology, Medical Faculty, Heinrich‐Heine‐University Düsseldorf Düsseldorf Germany; ^7^ Department of Gastroenterology, Gastrointestinal Oncology, Hepatology, Infectiology, and Geriatrics University Hospital of Tübingen Tübingen Germany

**Keywords:** duodenal, jejunal, LCIG, LECIG, Parkinson's disease

## Abstract

**Background:**

Real‐world clinical evidence is missing to understand the resorption characteristics of levodopa through duodenal and jejunal parts of the small intestine.

**Objective:**

To characterize how different application sites of intestinal levodopa gel would impact on levodopa dosing and clinical outcomes.

**Methods:**

This multicentre retrospective analysis investigated Parkinson's disease patients (n = 111) and their change in levodopa equivalent dosage when switching from oral treatment to intestinal continuous infusion therapy while stratifying for differences in percutaneous gastrojejunostomy (PEG‐J) tube localizations. We analyzed data from patients treated with both levodopa–carbidopa (LCIG) and levodopa–carbidopa–entacapone (LECIG) intestinal gel.

**Results:**

In dichotomic analysis, duodenal and jejunal tube positions showed similar levodopa equivalent dosages changes from baseline (*P* = 0.143). This was similar when subdividing patients for LCIG and LECIG treatment. In duodenal PEG‐J positions, 44.4% of patients showed persistent motor fluctuations compared to 21.9% in jejunal placements (*P* = 0.026). In duodenal positions, fluctuations most often persisted when the PEG‐J tube was placed proximally into the duodenum. In jejunal localizations, several patients displayed a satisfactory outcome from the primary intervention but experienced dislocation of the PEG‐J tube to a duodenal position. This was associated with re‐emergence of motor fluctuations in a majority of them.

**Conclusions:**

Our real‐world data suggest that LCIG and LECIG are absorbed similarly in both duodenal and jejunal portions of the small intestine. However, clinical data suggest, that jejunal positioning is critical to the stabilization of dopaminergic motor fluctuations.

Continuous intestinal infusion of levodopa‐containing gel into the small intestine has evolved as a standard therapy to mitigate the short‐comings of oral pulsatile replacement therapy in Parkinson's disease (PD). Several studies demonstrated that intestinal infusion of levodopa–carbidopa intestinal gel (LCIG)^1^, ^2^, ^3^ and levodopa–carbidopa–entacapone intestinal gel (LECIG)^4^, ^5^, ^6^ achieve stable plasma concentrations with lower variation coefficients compared to oral treatment.^7^

It is common to attempt the endoscopic placement of a percutaneous gastrojejunostomy (PEG‐J) tube distal from the ligament of Treitz (LoT). While this conceptual thinking is mainly based on the assumption that levodopa resorption would largely occur in the proximal jejunum, clinical evidence to substantiate this assumption remains scarce.^8^, ^9^ In contrast, other work stated that levodopa resorption would occur in the entire duodenum and proximal jejunum^10^ through large neutral amino acid transporters (LNAA). Again, these assumptions lack robust clinical real‐world evidence and were based on an early intestinal infusion study,^11^ or a transcriptomic expression study of the LNAA.^12^ More recent modeling work estimated that levodopa is being absorbed equally across all parts of the small intestine while other amino acids are only absorbed in the proximal jejunum.^13^, ^14^

Pharmacological considerations add to this framework. Once levodopa has entered the gastrointestinal tract, enzymatic pathways located both in the gastric and small intestinal wall convert levodopa to dopamine (before enteric resorption) by aromatic L‐amino acid decarboxylase (AADC) and to a lesser extent to 3‐*O*‐methyldopa by catecholamine‐*O*‐methyltransferase (COMT),^15^, ^16^, ^17^ and this peripheral metabolism is attenuated by both the dopa decarboxylase inhibitor carbidopa (LCIG, LECIG) and the COMT‐inhibitor entacapone (LECIG). Topographically, the exact distribution patterns of these enzymes within the duodenum and jejunum remained inconsistent with previous studies.^17^, ^18^

There are practical considerations to favor PEG‐J tip localization distal from LoT. A more proximal position could lead to reflux of the intestinal gel into the stomach leading to lower resorption, higher intraluminal metabolism through AADC and COMT enzymes, and therefore—potentially—more unstable clinical response patterns. These practical considerations are of major importance given that numerous high‐quality studies reported an unacceptably high rate of dislocations of the PEG‐J tip in conventional technique.^8^ However, in real‐world clinical practice the desirable tip position distal from LoT is not easily achieved.^8^

While all these aspects are relevant to clinical decision‐making and patient management, evidence from real‐world clinical practice is missing. To understand, whether distinct portions of the duodenum and jejunum differ in levodopa resorption and degradation, as well as in long‐term clinical outcomes is therefore important. We set up this retrospective multicentre analysis of several German expert centres to estimate whether PEG‐J tube position affects patients’ levodopa demand. As a proxy, we analyzed the change of post‐interventional levodopa equivalent dosages (LED) from pre‐interventional levodopa‐equivalent dosages and stratified for PEG‐J localization. Further, we evaluated long‐term clinical outcomes of patients depending on tube position within the first year from primary intervention.

## Methods

**Figure 1:**
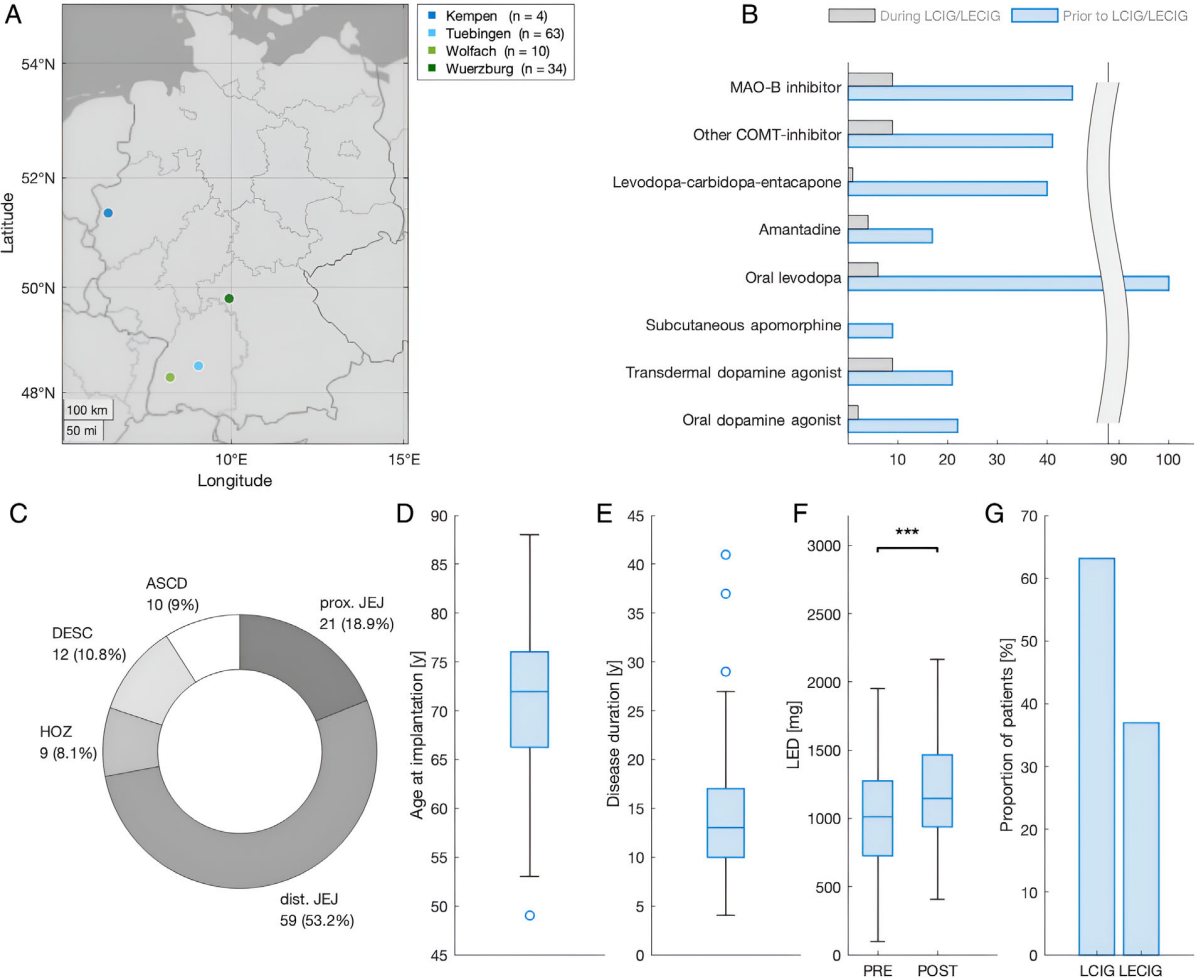
Demographic and clinical descriptives for the entire cohort of included subjects (N = 111). (**A**) Map of participating centers throughout Germany listed from east to west; size of the bubble depicts the number of contributing cases. (1) Hospital zum Heiligen Geist, Department of Neurology and Neurorehabilitation, Kempen; (2) Parkinson‐Klinik Ortenau, Wolfach; (3) University Hospital Tuebingen, Centre for Neurology, Tuebingen; (4) University Hospital Wuerzburg, Department of Neurology, Wuerzburg. (**B**) Treatment with other oral dopaminergic Parkinson's disease medication prior to (blue) and during (gray) levodopa carbidopa intestinal gel (LCIG)/levodopa carbidopa entacapone intestinal gel (LECIG) therapy. (**C**) Donutchart depicting intestinal tube locations; ASCD, duodenum ascending part; DESC, duodenum descending part; HOZ, duodenum horizontal part; prox. JEJ, proximal jejunum; dist. JEJ, distal jejunum. (**D**) Age at implantation (years). (**E**) Disease duration at time of implantation (years). (**F**) Levodopa equivalent dose in mg (LED) calculated before (PRE) and after initiation of intestinal infusion therapy (POST). (**G**) Proportion of patients treated with LCIG and LECIG therapy; *** denotes statistical significance at *P* < 0.001.

This study retrospectively reviewed the clinical records of patients with idiopathic PD who underwent implantation of an intestinal infusion pump at four different German expert centers between January 1, 2009 and March 1, 2024 (Fig. 1). The Ethics Committee of the University of Tübingen reviewed and approved the study protocol (839/2022BO2). Each site's Ethics committee independently approved the study. All data were obtained retrospectively as part of routine clinical records. According to the existing German law and judgment of the local data protection officers, no informed consent was required.

### Patient Selection

We included data from adult PD patients (≥18 years of age) who were prescribed continuous intestinal dopamine replacement therapy under regular clinical care. Patients received one of the following two formulations at the discretion of their prescribing physicians: (1) Levodopa–carbidopa intestinal gel (LCIG; containing 20 mg/mL levodopa and 5 mg/mL carbidopa monohydrate solution; AbbVie Ltd., Chicago, IL) for use in a portable CADD pump (Smiths Medical, Minneapolis, MN), or (2) intestinal levodopa (20 mg/mL), entacapone (20 mg/mL) and carbidopa (5 mg/mL) (LECIG; Stadapharm GmbH, Bad Vilbel, Germany) through a portable pump (Cané, Rivoli, Italy).

Patients were excluded in case (1) there was no post‐interventional imaging data (abdominal X‐rays, X‐ray‐fluoroscopy) available to determine tube localization, (2) radiological tube location remained unclassifiable based on available imaging data, (3) patients had received unknown study medication if having been treated within an interventional clinical trial, (4) there was insufficient data for analysis, (5) the patient developed peritonitis/severe systemic infection after tube implantation, (6) if they had deep brain stimulation.

### Evaluation of Tube Localization and Treatment Parameters

As per standard of care, patients were admitted to hospital for placement of a PEG‐J tube. In general, the tip of the jejunal extension tube is intended to be placed distal of the LoT. However, clinical and procedural reasons may lead to a more proximal position. We analyzed patients in whom tube localization was evaluated radiologically by means of abdominal X‐rays or X‐ray‐fluoroscopy. Imaging was obtained in the first few days from primary intervention during the initial in‐hospital stay lasting around 7 to 14 days. In case, the initial position was considered inappropriate and followed by early PEG‐J revision during the initial stay, we used the latest image before discharge from hospital. After hospital discharge und during the first year from primary intervention, no regular imaging of PEG‐J localization was performed unless there was clinical suspicion of PEG‐J displacement. This is generally the case when previously improved motor fluctuations or uncontrolled off‐times re‐emerged.

The pump is commonly programmed to operate throughout daytime hours from 6 am to 10 pm, but the exact running time may have varied individually between 5:30 am to 11:30 pm Daytime treatment starts with a morning bolus and is followed by continuous infusion at up to three constant rates until treatment is discontinued in the evening. Thirteen patients received a 24‐hour treatment with a reduced overnight rate and omitting the morning bolus.

### Data Collection and Processing

We analyzed the following variables: demographic information (gender, age at diagnosis, disease duration, age at implantation), clinical descriptives (PD subtype, Hoehn & Yahr stage, MDS‐UPDRS‐III scores upon admission), medication both at admission and at hospital discharge that mostly occurred within the first 7 to 14 days from primary PEG‐J intervention depending on the centers’ routines. Further, we requested the post‐interventional imaging data to determine the individual tube localizations.

### Calculation of Levodopa Equivalent Dosages

Levodopa equivalent dosages for all oral medication including LECIG and LCIG were calculated using the most recent conversion factors as suggested by the International Parkinson and Movement Disorders Society Non‐Motor Parkinson Disease Study Group.^19^ We determined the LEDs (1) from the pre‐interventional medication chart and upon hospital discharge after primary intervention of intestinal treatment.

We determined LED administered throughout daytime hours. For harmonization, we defined “daytime hours” ranging from 6 am to 10 pm. Post‐interventional LEDs were evaluated based on the discharge medication using the hours of continuous rate, morning bolus, and any other additional oral/transdermal dopaminergic medication. Extra doses were not considered. In patients with 24‐hour treatment regimen, we analyzed the LED delivered between 6 am and 10 pm for the sake of comparison with the pre‐interventional daytime LED.

### Anatomical Localization of the PEG‐J Tube

**Figure 2:**
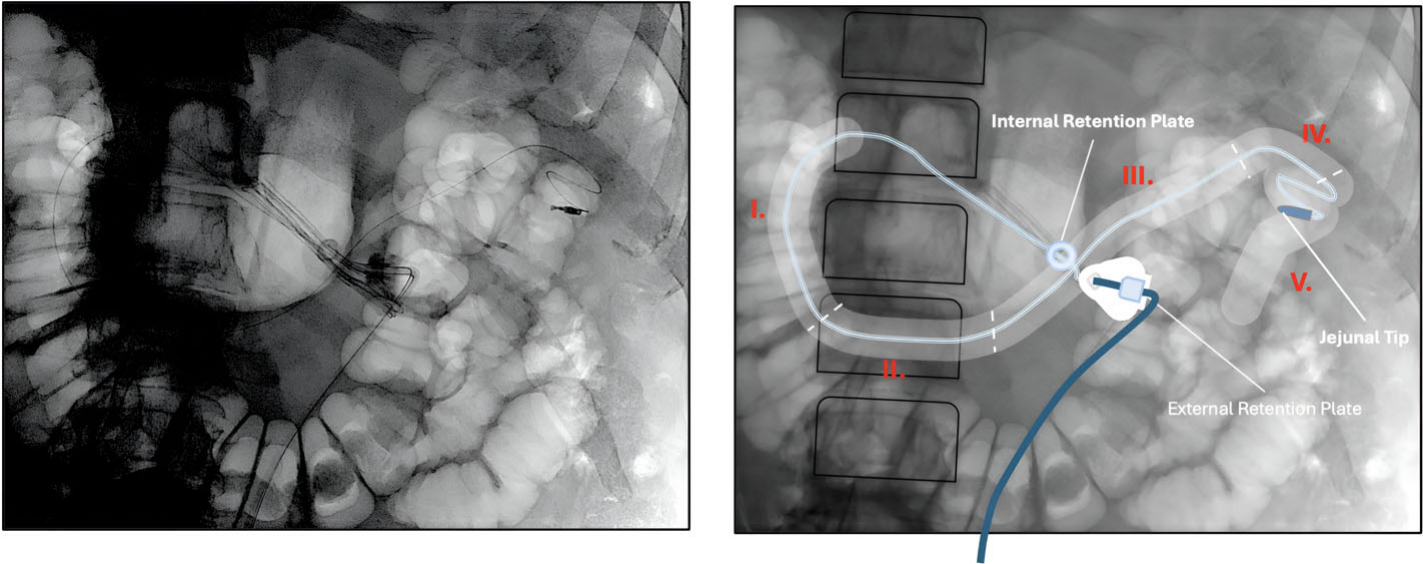
Exemplary schematic overview of duodenal and jejunal regions of interest based on X‐ray fluoroscopy (adapted from Berger et al^20^). The tip of the tube seen here is located in the distal jejunum (**V**.). Remaining intestinal sections were marked in roman numerals (red): (**I**.) Descending part of the duodenum (the tube descends right from the spine). (**II**.) Horizontal part of the duodenum (the tube crosses from the right side back to the left side of the spine). (**III**.) Ascending part (the tube ascends towards the Ligament of Treitz ‐ LoT). (**IV**.) Proximal jejunum (at the LoT and within 5 cm thereof; the tube is seen turning at least 90° with the midpoint of this turn regarded as the transition from the duodenum to the jejunum). (**V**.) Distal jejunum (the tip of the tube is located >5 cm distal of the LoT).

Percutaneous gastrojejunostomy tube placements were reviewed retrospectively based on X‐ray of fluoroscopy images. Localizations were determined from anatomical landmarks (adapted from Berger et al^20^) and stratified according to mutually exclusive anatomical positions: (1) descending part of the duodenum (DESC; the tube is seen descending right from the spine), (2) horizontal part of the duodenum (HOZ; the tube crosses from the patient's right side back to the left side of the spine), (3) ascending part of the duodenum (ASCD; the tip of the tube ascends towards the LoT), (4) proximal jejunum (prox. JEJ; at the LoT and within 5 cm thereof; the tube is seen turning at least 90°, and the midpoint of this turn can be regarded as the transition from the duodenum to the jejunum with the turn usually facing downwards) and (5) distal jejunum (dist. JEJ; the tip of the tube is located >5 cm distal of LoT) (Fig. 2). According to Berger et al, we defined the LoT as the transition point when the ascending part of the duodenum turns into the proximal jejunum, and this was considered to be reflected by the transition point when the tube shows a turn of at least 90°. Usually, this turn points downwards, but occasionally can show different spatial orientation (Berger et al^20^). In jejunal positions, distances from LoT and the tip were measured using a DICOM viewer in most instances. In a few patients we had to use a conventional image viewer and estimated the distance from the length of the tip, keeping in mind that this may yield some inaccuracy in particular when the jejunal catheter is oriented in a more anterio‐posterior position, distal from LoT in images with anterio‐posterior recording acquisition technique. In cases of uncertainty, an independent general radiologist was consulted to review the tube's position (n = 3). In case no consensus was achieved the image was labeled unclassifiable and withdrawn from further analyses (n = 2).

### Post‐Interventional Levodopa Equivalent Dosage

To approximate whether tube localization impacts on levodopa demand, we analyzed the relative change in LED between baseline (pre‐interventional LED) and post‐interventional LED expressed as percent change from baseline.

(eq 1) $$\left(\frac{{\mathrm{LED}}_{\text{post}-\text{interventional}}–{\mathrm{LED}}_{\mathrm{pre}-\text{interventional}}}{{\mathrm{LED}}_{\mathrm{pre}-\text{interventional}}}\times 100\right)$$

We stratified these metrics according to different tube localizations.

### Clinical Outcome of Patients with Duodenal and Jejunal Tube Localizations

We were interested to understand the clinical outcomes of patients within the first year from start of intestinal therapy. We subsequently requested the following information from the regular and narrative clinical records from the first year from primary intervention, whether: (1) patient was lost to follow‐up in the first year from intervention (“yes,” “no”); (2) there were persistent motor fluctuations after primary intervention; that is, fluctuations relevant to affect activities of daily living and unsatisfactory improvement based on patient's and clinician's subjective impression were considered persistent (“yes”), whilst relevantly improved or only mild and non‐bothersome residual fluctuations were considered relieved (“no”). This decision was made by one of the centers’ investigators (K.L.—Kempen; P.K.—Tübingen; C.D.—Würzburg; J.K.—Wolfach). In addition, we requested in patients with primary jejunal position and initially recovered fluctuations whether fluctuations re‐emerged in those presenting with PEG‐J dislocation, that is, dislocation was assumed in case an initial jejunal position was found in a duodenal or gastric position; (3) PEG‐J revision was performed (“yes,” “no,” “unknown”; date of revision); (4) cause for revision (“persistent motor fluctuations,” “disconnection of the PEG‐J tube from the connector,” “dislocation of PEG‐J tube,” “occlusion of PEG‐J tube,” “stoma infection,” “other”); (5) whether a jejunal position was achieved after the revision; and (6) in case a revision procedure took place, whether clinical outcomes were reported as improved based on the regular clinical documentation.

### Statistical Analysis

Descriptive statistics were presented as mean and standard deviation (SD) unless stated otherwise. The relationship between qualitative variables was assessed using Fisher's exact test or *χ* ^2^ while paired‐samples *t* test, independent samples *t* test or Wilcoxon signed rank test were used to compare means of quantitative variables, depending on the distribution of data. To assess the effects of tube location on LED percent change from baseline we used a one‐way analysis of variance (ANOVA). A two‐way ANOVA was used when testing for both the effects of treatment modality (LCIG, LECIG) and tube localization. Group means were compared by Tukey's post‐hoc test.

For all tests, assumptions of normality (Kolmogorov–Smirnov test) and homogeneity of variance (Levene's test) were evaluated. In an attempt to account for the possible influence of associated variables on the outcome variable (post‐interventional LED), a multiple linear regression model (MLR) was fitted. Variables entered into this model included disease duration, gender, tube location sites and pre‐interventional LED as independent variable. Statistical interaction for each variable was analyzed using MATLAB (version 2023b, The Mathworks, Inc., Natick, MA) and IBM SPSS Statistics, v29 (IBM Corp., Armonk, NY). Statistical significance was set at *P* < 0.05 (two‐sided). In addition to reporting individual *P*‐values, means, 95% confidence intervals, *R* ^2^, b‐coefficients, *F*‐test, η^2^ and ηp^2^ are provided as indices of effect size, where applicable.

## Results

Four German centers provided datasets from 111 patients with similar gender distribution (62 males, 49 females; *P* = 0.217). Of these, 70 patients were treated with LCIG, and 41 patients with LECIG. Demographic and clinical descriptives were similar in the LCIG and LECIG subgroups (Table 1). Absolute LEDs of the entire cohort increased at the post‐interventional (1173 ± 367 mg) compared to the pre‐interventional stage (1049 ± 41 mg; *P* < 0.001).

**TABLE 1:** Clinical and demographic characteristics

Parameter	Total (N = 111)	Intra‐intestinal distribution		*P* value
		Duodenal (n = 31)	Jejunal (n = 80)	
Age at implantation (yr), median ± IQR	72 (9.8)	72 (8.0)	71.5 (11)	0.97
Gender (female), n (%)	49 (44.1)	12 (38.7)	37 (46.25)	0.53
Age at disease onset (yr), median (IQR)	57 (12)	55.5 (12)	57 (12)	0.89
Disease duration (yr), median (IQR)	13 (7)	14 (6)	12 (8)	0.95
Hoehn & Yahr stage, n (%)				
2	6 (5.4)	1 (3.2)	5 (6.3)	0.82
2.5	5 (4.5)	2 (6.5)	3 (3.8)	
3	51 (45.9)	15 (48.4)	36 (45)	
3.5	4 (3.6)	2 (6.5)	2 (2.5)	
4	35 (31.5)	8 (25.8)	27 ((33.8)	
5	10 (9)	3 (9.7)	7 (8.8)	
Parkinson's subtype				
Akinetic‐rigid, n (%)	77 (69.4)	20 (64.5)	57 (71.3)	0.60
Mixed, n (%)	33 (29.7)	11 (35.5)	22 (27.5)	
Tremor dominant, n (%)	1 (0.9)	0 (0)	1 (1.25)	
MDS‐UPDRS III, median (IQR)^a^	38 (17.5)	36 (20)	38 (17.5)	0.66
LCIG, n (%)	70 (63.1)	11 (35.5)	59 (73.8)	0.0003
LECIG, n (%)	41 (36.9)	20 (64.5)	21 (26.2)	
Pre‐implantation levodopa use^b^ (mg), median (IQR)	1011 (547)	1050 (689)	1006 (518)	0.65
Post‐implantation levodopa use^b^ (mg), median (IQR)	1144 (540)	1154 (440)	1129 (619)	0.80
LED‐change from baseline (%), median (IQR)	18.2 (52.2)	26.8 (36.1)	13.5 (45.8)	0.24

Abbreviations: IQR, interquartile range; LCIG, levodopa carbidopa intestinal gel; LECIG, levodopa carbidopa entacapone intestinal gel.

^a^ MDS‐UPDRS III, Movement disorder society—Unified Parkinson's Disease Rating scale part III assessed in medical ON at admission.

^b^ Administered levodopa equivalent dose (LED) upon awakening until 10 pm; Statistical significance for continuous variables was assessed using the Wilcoxon signed rank test. For categorical outcomes, statistical significance was determined using Fisher's exact test or the Chi‐square test, as appropriate.

### LED Change Stratified by Duodenal Versus Jejunal Tube Localization

We first analyzed LED changes according to a binary classification, that is, tubes located either proximal (“duodenal”: DESC, HOZ and ASCD), or distal from LoT (“jejunal”: prox. JEJ and dist. JEJ). Relative LED change was numerically higher from baseline in duodenal compared to jejunal localization, but this did not reach statistical significance (duodenal: increase of 24.3 ± 39.1% vs. jejunal: 13.0 ± 34.2%; *P* = 0.143).

### LED Change Stratified by Tube Localization and Treatment Modality

**Figure 3:**
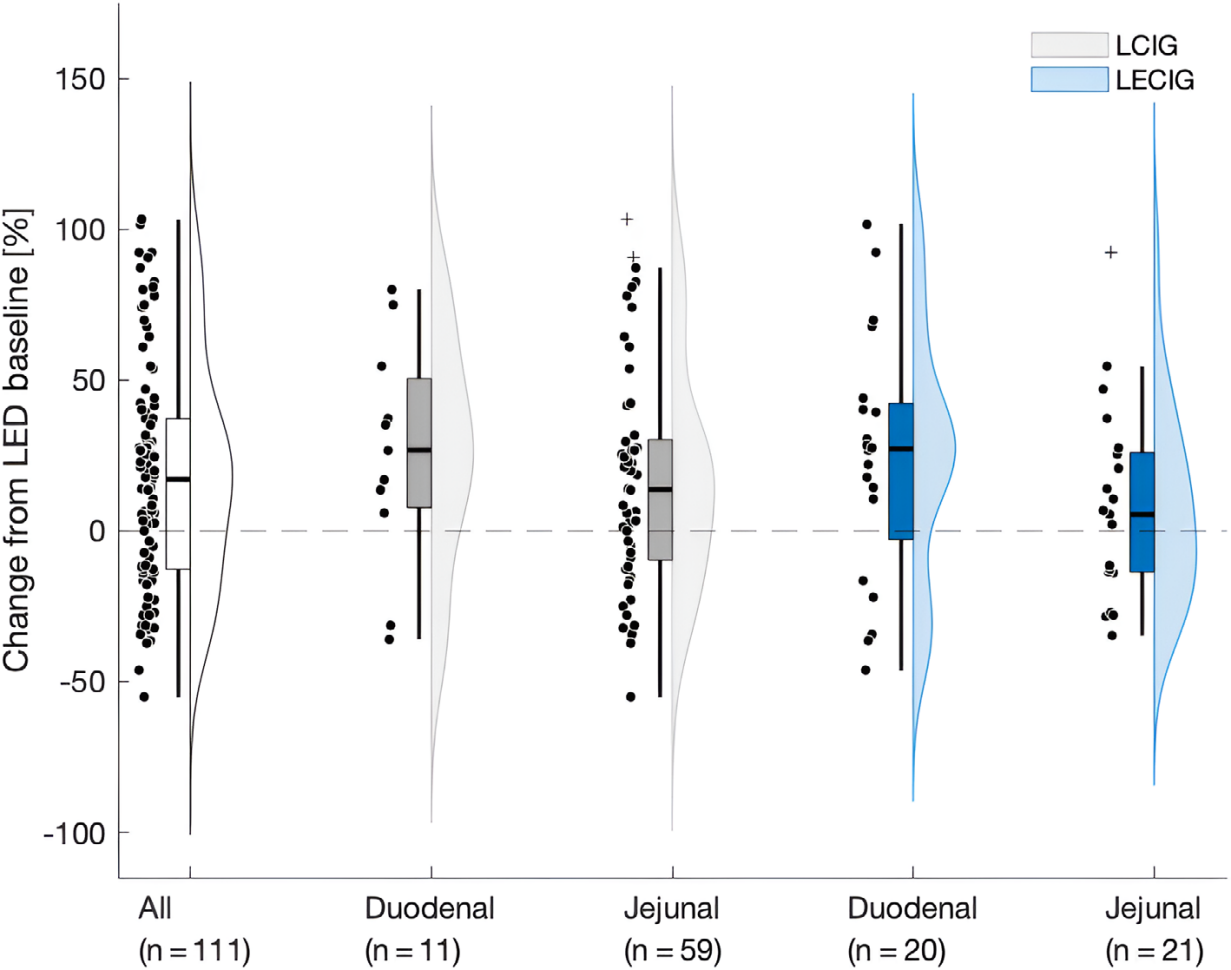
Relative percental change in LED from baseline stratified across different intra‐intestinal tube location sites and treatment modalities. LCIG, levodopa carbidopa intestinal gel; LECIG, levodopa carbidopa entacapone intestinal gel; LED, levodopa equivalent dose.

Further, we wished to control for the effect of intestinal COMT‐inhibition, and to evaluate whether relative LED percent change from baseline differed in LCIG compared to LECIG patients in the binary classification. In both groups, there was a numerically higher relative LED percent increase from baseline in duodenal compared to jejunal sites (LCIG: 25.2 ± 37.6 vs. 16.9 ± 36.8%; LECIG: 23.9 ± 40.9 vs. 7.5 ± 32.0%). However, the 2‐way ANOVA did not detect significant main effects for treatment modality (*F*(1,101) = 0.412, *P* = 0.523, ηp^2^ = 0.004) or tube localization (*F*(1,101) = 2.161, *P* = 0.145, ηp^2^ = 0.021). There was no significant interaction between treatment modality and tube localization (*F*(1,101) = 0.229, *P* = 0.633, ηp^2^ = 0.002) (Fig. 3).

### LED Change Stratified by Duodenal and Jejunal Sub‐Portions

To study whether the resorption characteristics depend on sub‐portions of the small intestine, we additionally stratified the cohort according to five mutually exclusive tube location sites (DESC, n = 12; HOZ, n = 9; ASCD, n = 10; prox. JEJ, n = 20 and dist. JEJ, n = 53). We performed this analysis on the entire cohort but did not subdivide by LCIG/LECIG treatment modality since this would have resulted in too small subgroups to warrant statistical evaluation.

**Figure 4:**
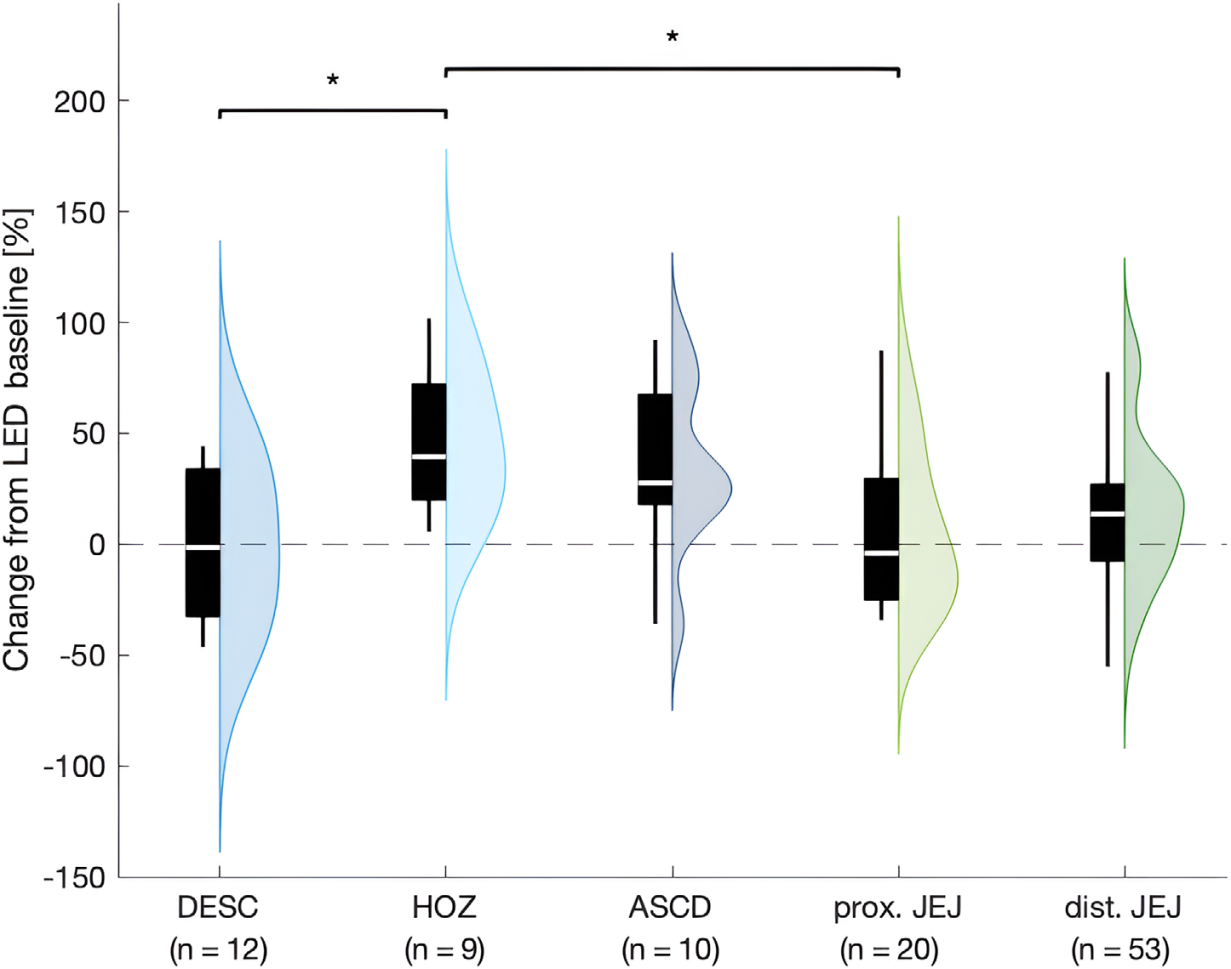
Relative percental change in LED from baseline stratified across different intra‐intestinal tube location sites. *Denotes statistical significance at *P* < 0.05; ASCD, duodenum ascending part; DESC, duodenum descending part; HOZ, duodenum horizontal part; prox. JEJ, proximal jejunum; dist. JEJ, distal jejunum; LED, levodopa equivalent dose.

There was a main effect of tube localization as determined by one‐way ANOVA [*F*(4,99) = 3.553, *P* = 0.009, η^2^ = 0.126]. All but two tube localizations showed a numerical percent LED increase from baseline. Tukey HSD post‐hoc tests revealed a significantly lower relative LED change from baseline in DESC (−0.37 ± 34.05%, *P* = 0.022) compared to HOZ (46.17 ± 32.54%) as well as in prox. JEJ (6.23 ± 35.59%) compared to HOZ (*P* = 0.036). No significant differences were found in the remaining localizations (dist. JEJ: 15.60 ± 33.62% and ASCD: 34.38 ± 36.55%) (Fig. 4).

### Clinical Outcomes of Patients with Duodenal and Jejunal Tube Localization

We reviewed the clinical records from the first year following intervention on patients with confirmed duodenal and jejunal tube localization. We were able to retrieve 100 records (duodenal: n = 27, jejunal n = 73) with at least one follow‐up, while 11 patients were lost to follow‐up.

From 27 patients with an initial duodenal tube position, 12 (44.4%) reported persistent motor fluctuations. Of these, seven were referred to interventional repositioning of the PEG‐J tube the same year (three of them after additional PEG‐J dislocation or disconnection had occurred). A jejunal position was achieved in three of these cases, improving the clinical outcome in one case. We further compared in patients with duodenal PEG‐J localization whether localization in sub‐portions of the duodenum differed in those with persistent motor fluctuations versus those with recovered motor fluctuations. Patients with persistent fluctuations showed a higher rate of proximal duodenal localizations DESC: n = 6 (50%), HOZ: n = 2 (16.7%), and ASCD: n = 4 (33.3%) compared to patients without motor fluctuations: DESC: n = 4 (26.7%), HOZ: n = 7 (46.7%), and ASCD: n = 4 (26.7%).

From 73 patients with a primary jejunal position, 16 (21.9%) patients reported persistent motor fluctuations after initial positioning of the PEG‐J tube. This unfavorable outcome occurred more rarely in patients with jejunal compared to duodenal position (*P* = 0.026). Of these, two patients received repositioning of the PEG‐J tube (resulting in one duodenal and one jejunal position) which did not improve the clinical outcome. Further, from patients with jejunal position in whom motor fluctuations were relieved after the primary procedure, 20 dislocations were reported in the first year from intervention. We found a close temporal relation of PEG‐J dislocation to a duodenal position with the onset of re‐emerging motor fluctuations in 10 patients. Further, there were 9 patients in whom re‐emerging motor fluctuations and PEG‐J dislocation were reported, but the exact temporal relation could not be retrieved from the records. There was one patient with PEG‐J dislocation and ongoing control of motor fluctuations thereafter. Overall, adverse events related to tube dislocation or disconnection were numerically higher, and statistically similar between jejunal PEG‐J tubes (34.2%) and duodenal tubes (18.5%; *P* = 0.128).

**Figure 5:**
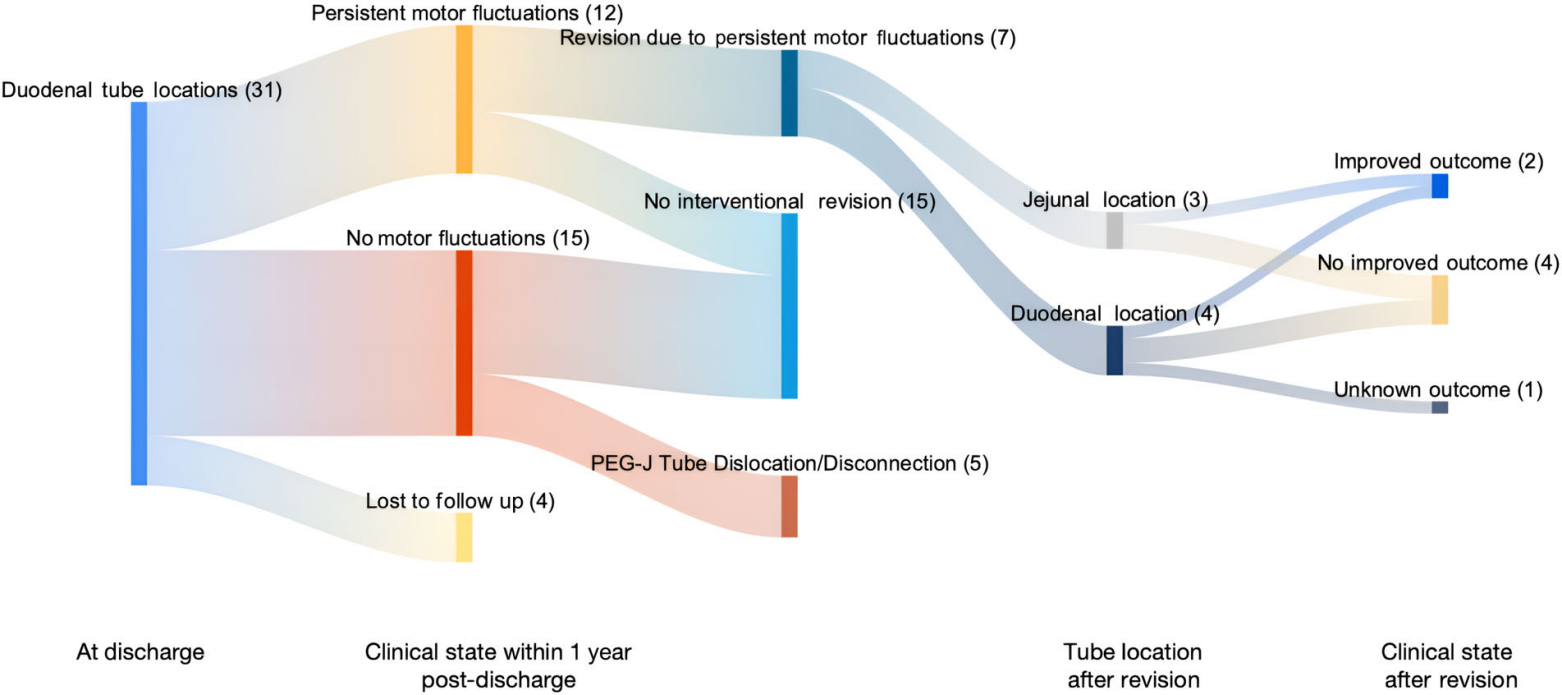
Clinical outcomes of patients with duodenal tube localization as assessed within 1 year after starting continuous intestinal infusion therapy displayed in a Sankey chart. Numbers of patients are given in parentheses.

**Figure 6:**
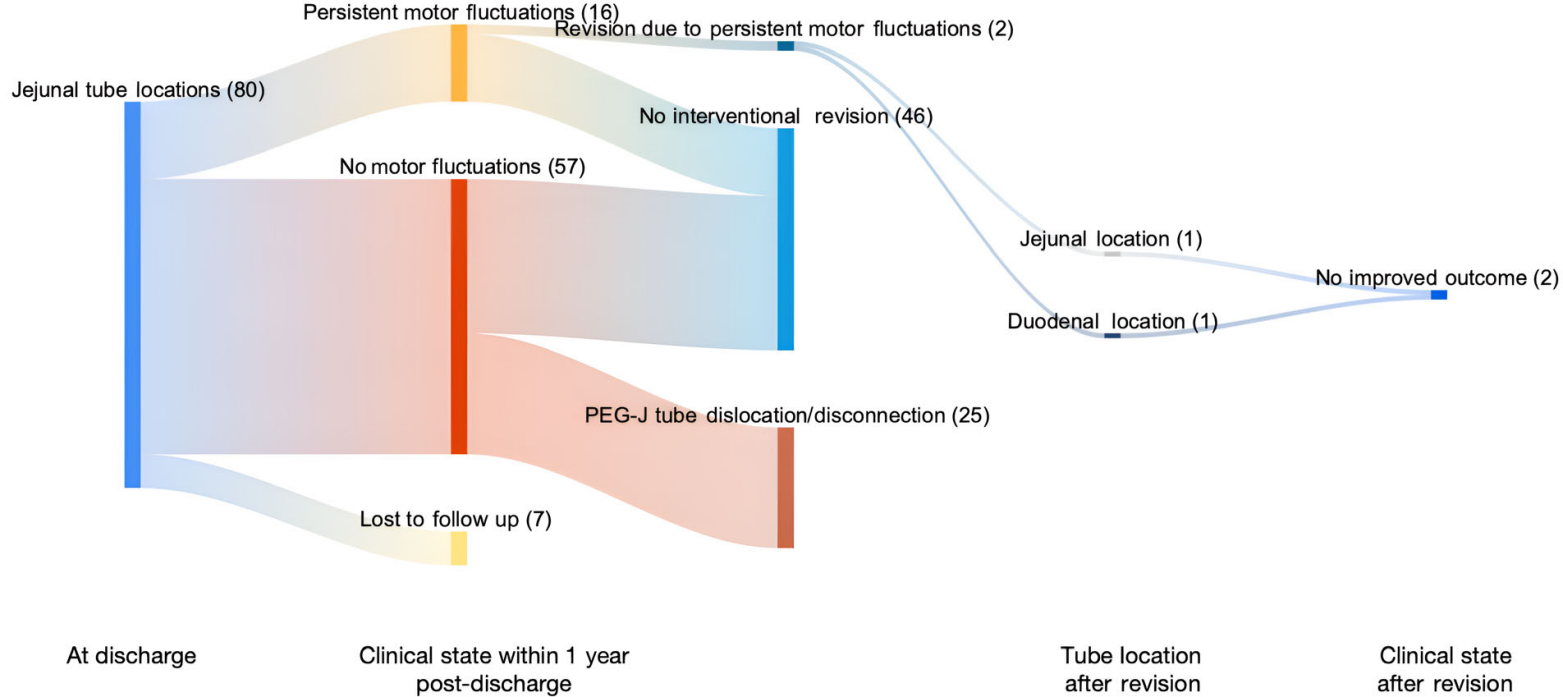
Outcomes of patients with jejunal tube localization as assessed within 1 year after starting continuous intestinal infusion therapy displayed in a Sankey chart. Numbers in brackets indicate number of patients.

A comprehensive overview of outcomes within 1 year following the tube's placement is provided as Sankey charts in Figures 5 and 6 for the duodenal and jejunal groups, respectively.

### Tube Localization Did Not Predict Post‐Interventional LED

Given the mean differences in relative change from baseline LED across different tube localizations, we fitted a MLR to assess the predictive value of tube localization on post‐interventional LED. The regression model accounted for approximately 41% of the variance in post‐interventional LED (*R* ^2^ = 0.412; adjusted *R* ^2^ = 0.368) and was suitable for predicting the outcome [*F*(7,94) = 9.419, *P* < 0.001] (Table 2). We found that both female gender and pre‐interventional LED were independent predictors of post‐interventional LED. Neither disease duration nor tube localization predicted the outcome variable.

**TABLE 2:** Multiple linear regression model (MLR) predicting post‐interventional LED

Predictor variable	Unstandardized coefficients		Standardized coefficients	T	*P*	(95% CI)
	B	SE	ß			
Constant	751.1	158.2		4.8	0.0000012	437 to 1065
Gender (male vs. female)	−149.1	61.0	−0.20	−2.4	0.016	−270 to −28
Tube location site						
Descending part	−156.1	99.7	−0.13	−1.6	0.121	−354 to 42
Horizontal part	80.1	107.9	0.06	0.7	0.460	−134 to 294
Ascending part	132.0	102.2	0.11	1.3	0.200	−71 to 335
Proximal jejunum Distal jejunum (reference)	−84.8	77.9	−0.09	−1.1	0.279	−239 to 70
Disease duration (yr)	7.1	5.7	0.10	1.3	0.213	−4 to 18
Pre‐interventional LED (mg)	0.53	0.09	0.53	6.1	0.000000015	0.36 to 0.70

Dependent variable = post‐interventional LED (mg); ANOVA: N = 102; *R* ^2^ = 0.412; adj. *R* ^2^ = 0.368; *F*(7,94) = 9.419.

Abbreviations: ANOVA, one‐way analysis of variance; B, unstandardized coefficient; ß, beta; CI, confidence interval; LED, levodopa equivalent dose; mg, milligram; *P*, *P*‐value; SE, standard error; *t*, *t* value.

## Discussion

This retrospective analysis is the first of its kind to characterize the relation between PEG‐J position, resorption characteristics of LCIG/LECIG, and clinical outcomes of motor fluctuations in the first year from primary intervention when switching oral therapy to intestinal levodopa infusion. We found that post‐interventional changes from baseline LED were similar in duodenal compared to jejunal placement, suggesting levodopa uptake in both the duodenum and the proximal jejunum. However, we found motor fluctuations less reliably improved in duodenal compared to jejunal position. This was substantiated by a higher rate of persistent motor fluctuations in patients with duodenal PEG‐J position. Further, dislocation of an initially well‐placed PEG‐J from the jejunum to a duodenal site led to re‐emergence of relevant motor fluctuations in 10 of 20 patients with confirmed dislocation.

### Similar Dosaging Characteristics in Duodenal and Jejunal Sites

There is an unresolved controversy, on whether levodopa is resorbed “only” in the jejunum, or whether there is relevant uptake in the duodenum as well. Our real‐world clinical data suggest that the resorption takes place both in the jejunum and the duodenum. If only resorbed in the jejunum, we would have expected an increase of LED in the duodenum compared to the jejunum, and higher equivalent dosages the more proximal a duodenal tip was placed. Further, reflux to the stomach might occur in more proximal duodenal positions, leading to an increase of LED. However, we did not find an increase in LED in more proximal positions. This finding is paralleled both by ancient case series^11^ and more recent considerations.^10^, ^12^, ^14^ Further, when dividing the duodenum into the descending, horizontal, and ascending parts, there should be a gradient with increasing dosages from the proximal to the distal duodenum. Longer duodenal transit time or reflux of levodopa intestinal gel to the stomach might then lead to higher levodopa degradation—but this was not the case.

LECIG and LCIG infusion offers clinically relevant insight on intestinal COMT metabolism in relation to intestinal tube localization. By inhibiting peripheral COMT, LECIG therapy allows to achieve equivalent plasma levels of levodopa at an approximately 30% lower dosage compared to LCIG to achieve bioequivalence.^5^ It has been inconsistently discussed whether COMT expression may show variation over the different portions of the small intestine,^17^, ^18^ and our findings on LECIG pre‐ and post‐interventional LEDs do not suggest a clinically meaningful difference. The findings stand in line with work on the specific neutral and dibasic amino acids exchanger (one specific transporter of the LNAA group relevant to levodopa absorption) in ex vivo preparations of a mouse model^21^ suggesting that levodopa uptake was similar across the duodenum and jejunum and not affected by concurrent administration of an AADC or COMT inhibitor.

Moreover, similar to previous LCIG/LECIG cohorts,^22^, ^23^, ^24^ our cohort showed a significant increase in LED after initiating continuous levodopa therapy, suggesting that patients under continuous levodopa therapy can tolerate higher daily doses of levodopa for optimal motor symptom control.^25^, ^26^

### Clinical and Practical Implications Concerning Tube Placement

Although duodenal and jejunal levodopa absorption seems to be comparable, clinical outcomes of the first year from treatment initiation pointed to meaningful differences of the subgroups. Close to half of the patients with duodenal localization exhibited persistent motor fluctuations which is well below the favorable group‐level outcomes from existing high‐quality trials^26^ and registries.^27^ These patients showed more often PEG‐J tube localization in the more proximal portions of the duodenum. The mechanisms of the persistent fluctuations may supposedly be a reflux of the levodopa gel to the stomach and may be based on the fact that the stomach does not express the LNAA transporters needed for levodopa absorption. Instead, the dopa decarboxylase enzymes degrade levodopa before it is transported to the small intestine. While still in the stomach, the typically prolonged gastro‐intestinal transit time—as in oral therapy—may be re‐established, and the advantage of continuous intestinal delivery wanes off. In line with these considerations, dislocation of an initially well‐placed tube from a jejunal to a duodenal position led to re‐emerging fluctuations in 10 of 20 patients.

Together, we argue that levodopa resorption occurs with both jejunal and duodenal PEG‐J tube positioning. However, proximal duodenal positions may yield a higher risk of persistent fluctuations. Our practical recommendation is to consider repositioning of a duodenal position depending on the clinical outcome. As the tube's position is only valid for the moment of X‐ray recording, re‐emergence of previously stabilized motor fluctuations may suggest PEG‐J displacement to the duodenum or stomach and should prompt radiological re‐evaluation of the PEG‐J position and eventually endoscopic or radiological revision. Clip‐fixation of the tube has been described^8^ and may be considered.

### Methodological Considerations

This retrospective analysis has several limitations. Data were collected from a wide timespan of more than 15 years. As such, data relating to post‐interventional outcomes like MDS‐UPDRS III or IV scores were available only in a minority of patients, and therefore we decided not to analyze them. Further, this retrospective study could not control for various factors that may impact on intestinal levodopa resorption and treatment outcomes. These include procedural aspects of the endoscopic intervention, body weight, genetic architecture (eg, COMT polymorphisms),^9^, ^10^ and the post‐interventional levodopa equivalent dosage may not have fully stabilized 7 to 14 days after treatment initiation of intestinal therapy. Further, consumption of high protein or dairy products, as well as gastrointestinal motility, helicobacter pylori infection, and small bacterial intestinal overgrowth may reduce intestinal levodopa absorption, and should be considered in case inappropriate PEG‐J position and dosage adjustments would not improve the clinical outcomes, in particular persistent off‐states.^28^, ^29^ Finally, when studying the longitudinal clinical outcomes, we had to rely on narrative information from the patient records. In particular, the dichotomous judgment on whether fluctuations persisted (“yes” or “no”) fell short of a standardized operational classification (like eg, MDS‐UPDRS IV or other fluctuation scale cutoff scores) but was based on the subjective patients reporting and clinician impression along real‐world routine clinical care. Nevertheless, we felt that this global clinical impression is relevant to clinical decision making—perhaps even more than scores from a formal fluctuation scale—when deciding on whether or not to reposition a duodenal tube position. Further, the findings generate important hypotheses that may be validated in future prospective and standardized clinical studies.

Our retrospective analysis provides real‐world clinical evidence that PEG‐J localization is of importance to achieve optimal treatment outcomes and a major aspect of troubleshooting in patients with suboptimal outcomes.

## Author Roles

(1) Research project: A. Conception, B. Organization, C. Execution; (2) Statistical Analysis: A. Design, B. Execution, C. Review and Critique; (3) Manuscript Preparation: A. Writing of the first draft, B. Review and Critique.

P.K.: 1ABC, 2ABC, 3AB.

M.L.: 1AC, 3B.

I.C.: 1C, 3B.

K.E.G.: 3B.

C.D.: 1C, 3B.

J.V.: 3B.

J.K.: 1C, 3B.

W.J.: 3B.

K.L.: 1C, 3B.

L.W.: 3B.

C.R.W.: 1C, 3B.

D.W.: 1A, 2C, 3AB.

## Disclosures

**Ethical Compliance Statement:** The Ethics Committee of the University of Tübingen reviewed and approved the study protocol (839/2022BO2). Each site's Ethics committee independently approved the study. Informed patient consent was not necessary for this work. We confirm that we have read the Journal's position on issues involved in ethical publication and affirm that this work is consistent with those guidelines.

**Funding Sources and Conflicts of Interest:** No specific funding was received for this work and the authors declare that there are no conflicts of interest relevant to this work.

**Financial Disclosures for the Previous 12 Months:** P.K. received honoraria as speaker from Orbit Health. KEG received honoraria as a scientific advisor for Fresenius‐Kabi. C.D. received honoraria as speaker and consultant from Abbvie. J.V. received honoraria as speaker and consultant from Medtronic, Boston Scientific, Abbott, Newronika, and Ceregate and as speaker from AbbVie and Zambon. W.J. received honoraria as speaker and/or consultant from Abbvie, Bial, Stadapharm, Zambon. L.W. reports to own stock in company BioNTech SE. He is consultant to the following companies or received travel honarium from: TEVA, UCB Schwarz, Desitin, Medtronic, Abbott/Abbvie, MEDA, Boehringer I, Storz Medical, Kyowa Kirin, Guidepoint, Merck, Merz, Synergia, BIAL, Zambon, Sapio Life, STADA, Inomed, Vertanical. D.W. received honoraria as speaker, consultant, and research grants from Abbvie, Abbott, Bial, Boston Scientific, Medtronic, Kyowa Kirin, and Stadapharm. All remaining authors declare that there are no additional disclosure to report.

## Data Availability

The data that support the findings of this study are available on request from the corresponding author. The data are not publicly available due to privacy or ethical restrictions.
